# The impact of nature‐based education on neurodivergent adolescent mental health and wellbeing: A systematic scoping review

**DOI:** 10.1002/jcv2.70182

**Published:** 2026-10-18

**Authors:** Helen Eke, Suzie Holt, Helen Dodd, Faraz Mughal, Eleanor Shaw, Abigail Emma Russell

**Affiliations:** ^1^ Department of Public Health and Sports Science University of Exeter Medical School University of Exeter Devon UK; ^2^ School of Medicine Keele University Keele UK; ^3^ Oxford Primary Care Clinical Trials Unit Nuffield Department of Primary Care Health Sciences University of Oxford Oxford UK

**Keywords:** adolescence, mental health, nature‐based education, neurodivergent, prevention, SEND, wellbeing

## Abstract

**Background:**

Around 10% of young people have a diagnosed neurodevelopmental condition or consider themselves neurodivergent (brain function different to that considered typical). Neurodivergent young people (NYP) utilise health services more frequently than their peers and are at increased risk of mental health problems, school absenteeism and suspension. Research shows that nature benefits mental health; nature‐based education (NBE) may be beneficial to NYP, providing opportunities for improved social skills, positive relationships, increased concentration and motivation outside traditional classrooms. Improved attendance, engagement with schooling, alongside improved support and social inclusion, will likely result in improved physical and mental health for NYP. This systematic scoping review aimed to provide an overview of research related to the impact of NBE on NYP mental health and wellbeing.

**Methods:**

Four electronic databases (MEDLINE, APA PsycINFO, Education Resources Information Centre, Education Research Complete) and supplementary grey literature were searched on 18/09/24 and 31/07/26 and reported according to the PRISMA‐ScR checklist. Eligibility criteria included NYP aged 10–19 years, NBE, mental health impact, English language, from 1990 to date. Data were extracted, charted descriptively, and synthesised narratively.

**Results:**

5767 records were screened, 97 at full text. Ten records (of nine studies) met inclusion criteria. Studies were heterogenous, small, exploratory, largely qualitative, most reported on Forest School and lacked generalisability. Mental health impact was often not the main objective and therefore not robustly captured, but all studies linked NBE to direct and indirect wellbeing benefits, including providing space and enhanced opportunities for social, cognitive, emotional, and motivational development and psychological resilience mechanisms.

**Conclusion:**

Research indicates the positive impact of NBE on mental health. Within the context of increasing nature deficit and worsening mental health for NYP, more work is needed to robustly evaluate NBE as an alternative educational model and identify key components that confer mental health benefits.

## Key Points

What's known?

- Nature‐based education (NBE) encompasses nature or outdoor practices primarily concerned with education or learning objectives.
- Current evidence base is limited and typically focuses on younger age groups; there is a significant evidence gap around NBE and neurodivergent young people (NYP).

What's new?

- Mental health impacts of NBE are not robustly captured in existing literature. Of note, all studies included in this review reported positive wellbeing effects, including providing space and enhanced opportunities for social, cognitive, and emotional development.

What's relevant?

- This review identifies a new avenue of research that is relevant across health and education spheres.
- Future research should evaluate NBE as an alternative educational model, and to identify key components that confer mental health benefits for NYP.

## INTRODUCTION

At least 10% of young people (YP) aged 10–19 years (‘adolescence’ as defined by the World Health Organisation) (World Health Organisation, 2025) are neurodivergent or have neurodevelopmental conditions (Francés et al., 2022a). Neurodevelopmental conditions are those which produce impairments in personal, social, academic or occupational functioning and can be defined by clinical criteria (Pike, 2024; Shah et al., 2022) and neurodivergent refers to people whose brains develop or operate differently to what is considered typical, based on a broader framework of cognitive function which includes traits and/or diagnosed conditions (Shah et al., 2022; The Open University, 2026). Neurodivergent young people (NYP) utilise health services more frequently than those who are neurotypical and are at increased risk of additional mental health problems in adolescence, relative to their peers (Arim et al., 2017; Siebald et al., 2016). The cost to the health service of treatment for NYP is estimated to be around four times higher than that of a young person without a neurodevelopmental condition (Holden et al., 2013). Existing first‐line NHS treatments for mental health difficulties are typically designed for neurotypical young people and may be a poor fit for treating conditions experienced by NYP (Alexander et al., 2021; Pinals et al., 2022) and so management of mental health difficulties places a large burden on primary, community and social care.

School attendance is a policy priority (Long & Roberts, 2024) as schools provide important opportunities for academic, social, and emotional development. YP with higher attendance achieve higher English and Maths GCSEs (General Certificate of Secondary Education, school examinations taken at age 16) with 84% of regular attenders achieving high level GCSEs, compared to 36% of those who were persistently absent; and schools can provide the opportunity for the formation of positive peer relationships, which is a critical mechanism for mental health disorder prevention (Children's Commissioner's Office, 2023; Department for Education, 2022). NYP have higher rates of school absenteeism than their neurotypical peers and are much more likely to be suspended from school due to disruptive behaviours, physical and somatic symptoms, school refusal, and family and peer problems (Bennett et al., 2022; John et al., 2022). Improved attendance and engagement with schooling (in any form), alongside improved support and social inclusion, is likely to result in a lower burden of adult mental health problems, improved functioning, better relationships, and improved physical and mental health for NYP (John et al., 2022). There is therefore a need to understand how we can provide NYP with inclusive and accessible provision, outside of and within mainstream education. Government policy in England supports provision of alternative education for all children appropriate to their age, ability and special educational needs and/or disabilities (SEND), emphasising that any alternative education should address the child's physical and mental health needs as well as their educational needs (Department for Education, 2025).

Research has shown that time spent in nature offers mental health benefits (Garden & Downes, 2023; Gill, 2014; Maes et al., 2021; McCormick, 2017). Nature‐based education is a term used in the UK (Jani et al., 2023), US (Van Laan & Merrick, 2023) and worldwide contexts (IUCN, 2024) to encompass nature or outdoor practices which are primarily concerned with education or learning objectives. It differs to more traditional approaches to education which are primarily based indoors, with teacher‐directed instruction (Ernst et al., 2021). In recent years, Forest Schools (FS) are one example of a NBE approach that has been established across the UK, providing a nature‐based alternative or complementary educational provision model defined as an “inspirational process that offers…. young people…regular opportunities to achieve and develop confidence and self‐esteem through hands on learning experiences in a woodland environment” (Forest School Association, 2024). Whilst research on FS and NYP specifically is relatively limited, initial evidence suggests that NBE may be beneficial to NYP (Fan et al., 2023; Padesky & Padesky, 2026). YP with poor behaviour reportedly benefitted more than their peers from FS (Roe & Aspinall, 2011) and benefits such as increased engagement, social interaction, confidence, and reduced anxiety and stress have been reported for those with SEND (Slade et al., 2013). Previous work shows that self‐esteem, peer and teacher relationships, and ability to pay attention are some of the most important outcomes for NYP in relation to school (Roe & Aspinall, 2011), and the beneficial effects for YP's mental health are characterised as improved social skills, self‐esteem, self‐efficacy, belonging, positive teacher relationships, and increased concentration and motivation (Dabaja, 2022; Garden & Downes, 2023; Hepworth et al., 2025; O’Brien & Murray, 2007). All of these are critical areas of socioemotional skill development for NYP and are protective factors for mental health (Layous et al., 2014).

The current evidence‐base around NBE is limited and typically focuses on younger age groups (pre‐school or lower primary) and there is a significant evidence gap in relation to NBE and NYP. Given increasing rates of recognition and diagnosis of neurodevelopmental conditions (Russell et al., 2022; Underwood et al., 2022), and the documented school attendance issues that can occur for young people with neurodevelopmental conditions, there is an urgent need to develop improved models of educational provision for NYP to better support their education and mental health, and to reduce burden on health and social care services. This systematic scoping review (ScR) has been undertaken as part of a wider study exploring the potential for NBE to be a preventative public health intervention for NYP. This is a new avenue of research interest, with proposed benefits to both health and education spheres.

The aim of this systematic ScR is to provide the first summary of research related to the impact of NBE on the mental health and wellbeing of NYP aged 10–19 years. For the purposes of this review, we use the term NYP to include young people with and without a clinical diagnosis. The review has the following objectives.

1. To identify the types of NBE for NYP that are reported in the evidence base.
2. To explore the evidence base for mental health impacts for NYP who have attended a NBE setting.
3. To identify the components of NBE that confer mental health impacts to NYP, as described in the current evidence base.

## METHODS

A systematic ScR was undertaken, guided by the Joanna Briggs Institute (Peters et al., 2020) and the Cochrane Handbook (Higgins et al., 2024) and reported according to the PRISMA‐ScR checklist (Preferred Reporting Items for Systematic Reviews and Meta‐Analyses‐Scoping Review Extension) (Tricco et al., 2018). It was based on a protocol developed by the project lead, available through Open Science Framework (OSF) via the following link; https://osf.io/mbcrn/overview?view_only=248908c0b4c7489096d076b973589113.

### Search approach and strategy

Four electronic databases were selected indexing both mental health and education fields: MEDLINE and APA PsycINFO (via Ovid), Education Resources Information Centre (ERIC) and Education Research Complete (via EBSCO). A grey literature search using google.com supplemented this. Backwards citation chasing was applied to reviews identified. A pragmatic rather than exhaustive approach was taken given our aim to capture an overview of existing reports in an efficient timeframe. Table 1 shows the search strategy used in all databases.

**TABLE 1:** Search strategy used in electronic databases.

Nature‐based education	AB (“forest*” OR “nature*” OR “woodland*” OR “outdoor educa*”)
Health condition	AB (“mental” OR “wellbeing” OR “health” OR “depression” OR “anxiety”)
Population	AB (“neurodiv*” OR “autis*” OR “ADHD” OR “develop*”)
Age group	AB (“adolescen*” OR “young*” OR “teen*” OR “youth*”)

The search terms were applied to the abstract field and included relevant truncated terms identified by the research team within the following categories: NBE AND health condition AND population AND age group. The same search string was applied for the grey literature search, with the first ten pages (approximately 100 records) of results screened for relevance. The search strategy deliberately used these specific terms, as the intention of the review was to *only* identify reports that directly linked NBE to the adolescent neurodivergent population and mental health outcomes. The terms selected reflected this.

### Eligibility criteria

A PICOTS (population, intervention, comparison, outcome, timeframe) framework (Hosseini et al., 2024) was used. The final eligibility criteria are shown in Table 2. Peer‐reviewed English‐language publications or grey literature reports from any country from 1990 onwards were eligible if they incorporated the following: population aged 10–19 years (+/− 1 year; or if range not reported, mean age within 10–19 years); identified as neurodivergent, having a neurodevelopmental diagnosis, or having SEND or Social Emotional and Mental Health (SEMH) needs; involving a NBE provision; and exploration of mental health or wellbeing impact.

**TABLE 2:** Final inclusion and exclusion criteria.

Inclusion	Exclusion
Year 1990 to present day	Pre year 1990
English language	Non‐English language
Neurodivergent population (those with a neurodevelopmental condition e.g. autism or ADHD, or with SEND, or SEMH difficulties).	Neuro‐typical populations without a neurodevelopmental condition, SEND or SEMH difficulties.
Population aged 10–19 years (+/− 1 year) or reported mean age within 10–19 years.	Population outside of 10–19 years (+/− 1) or reported mean age outside of 10–19 years.
Published research papers, reports or reviews, and grey literature, describing ALL of the following: Nature‐based education (defined as nature or outdoor practices primarily concerned with education or learning objectives; but not where main aim is psychotherapeutic or medical treatment or intervention) Mental health or wellbeing impacts	Research papers, reports or reviews on nature‐based education where the main aim was psychotherapeutic or medical treatment or intervention (e.g. Wilderness therapy). Research papers, reports or reviews on outdoor physical adventure (climbing, camping, canoeing etc.). Research papers, reports or reviews with no report of mental health or wellbeing impacts Conference abstracts

All study types, theoretical perspectives, primary or secondary analyses, review articles, project reports, and opinion pieces were eligible. A date limit of post‐1990 was selected because availability of NBE and concepts of alternative education are a relatively recent phenomenon. The most utilised example of NBE practice in the UK, FS, was brought to the UK in 1993 (Cree & McCree, 2012). The age range of 10–19 was selected as this reflects the definition of adolescence as defined by the World Health Organisation (World Health Organisation, 2025), and only studies in English were included as there were no translation resources available to the research team.

The criteria aimed to encompass all neurodevelopmental disorders, however only Autism and ADHD were specified in the search strategy as these are reported as being the most commonly identified and studied neurodevelopmental disorders in the adolescent population (Francés et al., 2022b). There is limited consensus on which diagnoses are included within the term neurodivergent, and it is acknowledged that comorbidity of multiple neurodevelopmental disorders is the norm (Bellato et al., 2026; Megan Cleaton & Kirby, 2018), and hence our search strategy included truncated terms including ‘*neurodiv’* and ‘*develop’*, and mental health conditions such as ‘*anxiety’* and ‘*depression’* to ensure studies that incorporated a wide range of diagnoses were identified.

The descriptor, *NBE*, is an umbrella term used in the UK (Jani et al., 2023), US (Van Laan & Merrick, 2023) and worldwide contexts (IUCN, 2024). For the purposes of this review, this was defined as nature or outdoor practices which were primarily concerned with education or learning objectives; but not where the main aim was psychotherapeutic or medical treatment or intervention, such as substance abuse interventions, an example of which is the US mental health treatment Wilderness Therapy (Tucker et al., 2016) where the outdoors functions to separate individuals from cues and provide extreme challenge (Fleming, 2022). Outdoor physical adventure (climbing, camping, canoeing etc.) was also excluded as it is a discrete leisure sector and not primarily education focused.

### Screening procedure

The searches were run on 18/9/2024 and updated on 31/07/26. CADIMA, a free web‐based tool for supporting literature reviews developed by the Julius Kühn‐Institut (Unger et al., 2023) was used to facilitate screening. Firstly, reports identified in databases were imported into CADIMA, and duplicates detected and removed using its inbuilt functionality. Secondly, two researchers (HE/ES, and HE/MM) independently screened title and abstracts and discrepancies were discussed. Thirdly, full reports were independently screened by two researchers (HE/SH and HE/MM) with further meetings to resolve discrepancies.

### Data charting

Data extraction included descriptive information on study details, setting, population, design and intervention characteristics. Findings were extracted as numerical data and qualitative themes as recommended (Arksey & O’Malley, 2005) to synthesise information on mental health impact and ‘active’ components (review objectives two and three). A pre‐specified data charting sheet was developed to provide structure and enhance objectivity, based on the PICOTS framework. Three researchers (SH, ES and HE) independently extracted data from each report in order to minimise errors (Higgins et al., 2024). Only directly relevant data were extracted for example where differentiable, findings pertaining to ages outside the specified range were not included. All relevant findings were mapped and summarised descriptively in the spreadsheet. HE oversaw screening, data extraction, and researcher discussions to resolve discrepancies.

Findings from the extracted data were narratively described (Popay et al., 2006) to address the three objectives on types of NBE, mental health impact and nature‐based components, but also to provide contextualised understanding (including study types, overarching theory and nature of samples). To address objective one a PICOTS‐structured evidence table was developed to summarise the types of NBE provision. Analysis also involved frequency counts of provision type and intervention characteristics (type, aims, site, practitioners, adult‐child ratios, frequency and duration), as well as examination of populations sampled. To address objectives two and three, a summary table outlined relevant high‐level findings. As well as narrative description, qualitative content analysis was selectively deployed (Peters et al., 2022), to provide categorisation of similar themes relating to wellbeing impact across heterogeneous studies. Carried out inductively, relevant data from the extraction spreadsheet was listed, possible categories were noted, and data were revisited several times to assess best fit. A formal quality assessment procedure was not undertaken, as this was beyond the remit of a ScR (Peters et al., 2022; Pollock et al., 2022). Instead, relevant quality limitation factors noted by study authors, along with supplementary comments by the review team, are considered in the discussion.

## RESULTS

A total of 7401 records were identified from the database search (Figure 1). Following removal of duplicates, 5624 records were screened at title/abstract stage. Of these, 5507 records were excluded for not meeting criteria, and two could not be retrieved. 115 reports were assessed for eligibility at full‐text stage. In addition, 23 reports were identified through the grey literature search and via backward citation searches of two reviews (97 reports retrieved in full). Ten reports (reporting on nine studies) met the criteria and were included in the review. For the two reports documenting the same study (Tiplady, 2018; Tiplady & Menter, 2022), information was drawn from both papers as required to answer our research objectives.

**FIGURE 1:**
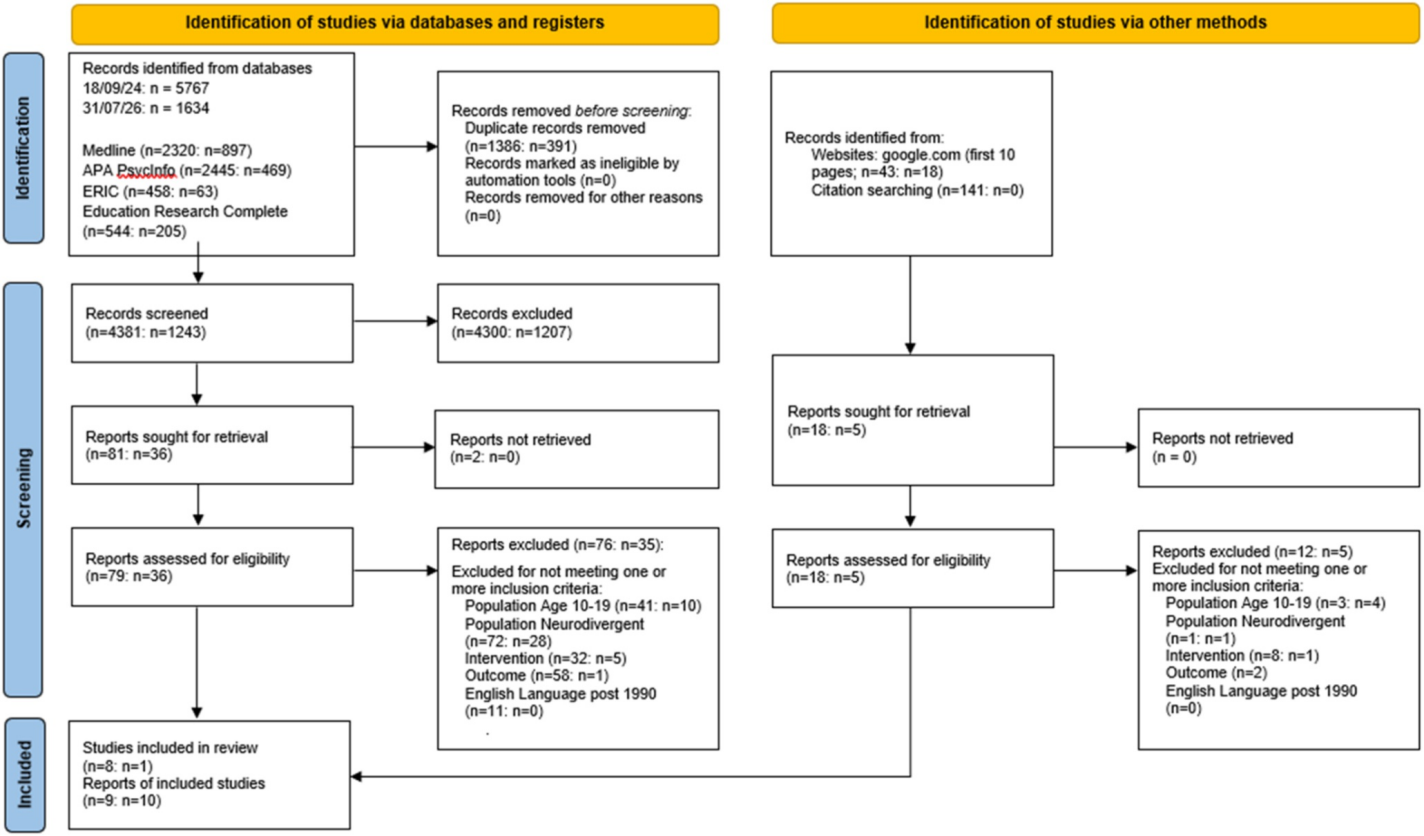
PRISMA 2020 flow diagram for database searches and other sources. Many reports were excluded on multiple criteria, resulting in exclusion frequencies that total greater than the total number of studies.

### Key characteristics of studies

Of the nine included studies, all comprised small‐scale peer‐reviewed primary research. Three used mixed methods analyses (Chiumento et al., 2018; Garip et al., 2021; Tiplady & Menter, 2021); four were qualitative (Friedman et al., 2024; Manner et al., 2021; Saggers & Strachan, 2016; Tiplady & Menter, 2022), and two quantitative (Roe & Aspinall, 2011; Tonks et al., 2025). Tiplady and Menter (2022) described a near replication of their 2021a paper (Tiplady & Menter, 2021), in that the same intervention, site and qualitative analysis was applied to a new sample in the subsequent calendar year. Sample characteristics were heterogeneous. Overall, sample size was small (range 7–54; Median = 19.7), with quantitative samples ranging from 7 to 54 (Median = 24). See Table 3 for individual study details.

**TABLE 3:** Table of key characteristics of included studies.

Citation	Population (within scoping review age range^a^)	Intervention	Comparator/Control	Outcomes used	Study type	Timeframe
Chiumento et al. (2018)	Participants ( *n* = 36): Pupils (9–15 years) with social, emotional and behavioural difficulties. Settings: Mainstream mixed secondary and primary schools (Yrs 5–9) in deprived neighbourhood in North West England.	Social and therapeutic horticulture (*Haven Green Space STH*): a school gardening intervention incorporating psycho‐therapeutic techniques. Aimed at promoting emotional wellbeing and mental/physical health using *Five Ways to Wellbeing* framework (Aked et al., 2008) Site: School grounds. Practitioners: 2 horticulturists and a psychotherapist (ratio of ∼3 adults to 12 pupils).	Within‐participant: Pre‐post mental wellbeing impact assessment and wellbeing check scores (start and end of 6‐month study). No control group.	Qualitative: Mental Well Being Impact assessment prioritisation grid (MWIA collaborative, 2011). Quantitative: Wellbeing Check Cards (WEMWBS^1^, university of Warwick 2006). N.B. Teacher SDQs and interviews not reported.	Mixed methods exploratory pre‐post evaluation (to assess feasibility of intervention; suitability of evaluation; and identify changes in wellbeing and perceived impact on wellbeing factors).	Study length: 6 months. Frequency of sessions: Monthly: 6x 2‐h sessions.
Friedman et al. (2024)	Participants ( *n* = 25): Pupils with autism (8–11 years; *M* = 9.8 yrs). (NB *n* = 25 observed; *n* = 9 pupils interviewed and *n* = 10 parents interviewed). Setting: Autism specialist school in East England.	Forest School sessions as part of school timetable (FSA^2^ accredited). Site: Wooded Forest School are in school grounds. Practitioners: Level 3 Forest School Leader, Forest School assistant and school staff (ratio of up to 5 staff to 3–10 pupils)	N/A	Qualitative: Child observation; and parent and child semi‐structured interviews. Observations (field notes) repeated weekly at Forest School (and in indoor academic lessons to supplement insight).	Ethnographic observational case studies	Study length: 3–6 months. (Observation over 1 term; child interview at ∼ 6 months). Frequency of sessions: Weekly (hrs not specified).
Garip et al. (2021)	Participants ( *n* = 54): Young people (13–19 years) not attending school; at PRUs^3^; or unemployed (*n* = 25 with additional needs) from London. Setting: Hampstead Heath, London.	Outdoor education programme for teenagers (*Green Talent,* part of *Green Spaces, Learning Places (GSLP) initiative*). Structured activities to promote nature connection; environmental careers; resilience; confidence; and wellbeing. Site: Public green space (park, forest etc.). Practitioners: GSLP team: outdoor, school and play workers.	Within‐participant: self‐report before and after single session. No control group.	Qualitative ( *n* = 8): Structured interview after session (based on coproduced themes). Quantitative (n = 54): Bespoke self‐report survey at start and end of session.	Mixed methods, before and after study (including evaluation of intervention).	Study length: Single session (half or full day).
Manner et al. (2021)	Participants ( *n* = 8): Adolescent girls (12–13 yrs) with/at risk of poor mental health: Depression, anxiety, low self‐esteem. Setting: Forest School programme for girls at a mixed mainstream school in rural Scotland.	Forest School sessions as part of school day (based on Forest School ethos). Site: Local park woodland out of sight of school. Practitioners: Qualified Forest School Leaders (ratio of 4 leaders to up to 12 girls).	N/A	Qualitative: In‐depth semi‐structured interviews about experiences (during second block of sessions).	Qualitative interpretative observational study (to explore Forest School experiences around wellbeing, resilience and sense of community)	Study length: 3 month‐study (during a second 12‐week block Frequency of sessions: Weekly: 12x 3‐hr sessions
Roe and Aspinall (2011)	Participants ( *n* = 18): Pupils (*M* = 11 yrs; range nr) pre‐categorised into *good‐* (*n* = 6) or *poor‐behaviour* (*n* = 12, including pupils with ADHD; risk of exclusion; or ‘withdrawn’). Settings: Mixed secondary (*n* = 10) and boys' residential school (*n* = 8) (Yrs S1‐S2); in a deprived urban area of central Scotland.	Forest School sessions (no details reported). Site: Woodlands near the school. Practitioners: Not reported	Within‐participant: Before and after forest school session versus typical indoor school day. Between‐participant: *good‐* versus *poor‐ behaviour* groups (mood measure only).	Quantitative (validated measures): i. Mood ( *n* = 18): UWIST^4^ 14‐item mood adjective checklist (Matthews et al., 1990). ii. Personal projects reflection (*n* = 10): 6‐item scale adapted from little (1983) on personal goal and affect measures.	Quantitative observational pre‐post study (to assess psychological restoration and reflections across educational contexts and behavioural groups).	Study length: Single five‐hour session (1 week between school and Forest School time‐points).
Saggers and Strachan (2016)	Participants ( *n* = 11): Pupils (10–13 years) at risk of *school failure* or with social, emotional or behavioural difficulties. Setting: Mainstream primary in an area of low socio‐economic status in Australia.	Equine facilitated learning (EFL) focused on social‐emotional learning (during school). Needs‐led topics: self‐regulation; social connection/communication/relationships; relaxation; confidence; perseverance; planning; and achievement. Site: Riding school away from school site. Practitioners: Two EFL/PATH^5^‐trained leads; and two school staff (ratio of 1 adult: 2 pupils).	Within‐participant self‐report at pre and post intervention interviews.	Qualitative: In‐depth semi‐structured interviews (pre and post session block) examining prior perceptions and perceived achievement.	Qualitative pre‐post exploratory case study (to explore experiences and outcomes from intervention).	Study length: One term (8‐week block) Frequency of sessions: Weekly 8x 2‐h sessions).
Tiplady and Menter (2021) and Tiplady (2018) *(2018 reference is a publicly available pilot report for funders which is related to the same study reported in the 2021a paper. Information was drawn from both references to answer the research objectives)*	Participants ( *n* = 11): Pupils (12–13 years) with an EHCP^6^ and special educational needs (severe anxiety), the Forest School lead (*n* = 1), school staff (*n* = 2+) and parents/carers (*n* = 3). Setting: Independent‐run Forest School session with pupils from a specialist secondary school in a deprived area of Newcastle.	Forest School sessions (in school day, part of *Breeze Project*): Practitioner and school staff co‐designed and delivered bespoke sessions based on child‐need. Aimed at promoting emotional wellbeing and confidence through scaffolding task success, relationships and positive self‐talk. Site: Woodland within a community garden away from school grounds. Practitioners: Level 3 Forest School Leader/Trainer, with specialist schoolteacher and teaching assistant (ratio of 1 adult to 3 pupils).	Main analysis: N/A Additional analysis: Within‐participant emotional literacy measured pre‐post Forest School session.	Qualitative: *Pupils*: semi‐structured/participatory interview; diaries and observations. *Staff and parents/carers*: in‐depth interviews. *Other*: Fieldwork notes. Quantitative ( *n* = 5): GL emotional literacy self‐assessment scale (validated, standardised measure; Faupel, 2003) and attendance records	Mixed methods Qualitative case study (with quantitative pre‐post supplement). Main study: Participatory and ethnographic methods; and co‐produced mixed methods impact evaluation (of components producing impact on wellbeing.	Study length: Across a school year (2017–2018): From 6 months to a full year. Frequency of sessions: Weekly one‐day sessions.
Tiplady and Menter (2021)	Participants ( *n* = 8): Pupils (12–16 years) with an EHCP^6^ and special educational needs (social and emotional aspects linked to disability; trauma; or early adversity). Forest School Leaders, school staff and parents/carers (*n* = nr). Setting: As above (see Tiplady & Menter, 2021).	Forest School sessions based in FSA^2^ principles that is, learner‐centred, child‐led, skills scaffolding and relationship modelling. Site: Woodland within a community garden away from school grounds. Practitioners: Level 3 Forest School Leaders and school staff.	N/A	Qualitative: As above (see Tiplady & Menter, 2021).	Qualitative case study (participatory and ethnographic methods); and testing of a theory of change model (Tiplady & Menter, 2021) see above).	Study length: Across a school year (2018–2019) Frequency of sessions: Weekly one‐day sessions.
Tonks et al. (2025)	Participants ( *n* = 7) Key stage 3 students (11–14 years, all male) with special educational needs and EHCP. Setting: Special educational needs provision attached to a large comprehensive school in southwest England.	Modified 5‐week Northern Council for Further education (NCFE) level 2 Bushcraft activity programme to evaluate if intervention is feasible, acceptable and effective at improving mental health and self‐concept. Activities centred around site knowledge, shelter building, fire skills, wilderness living and campfire cooking. Site: Woodland away from school grounds Practitioners: Unspecified.	N/A	Quantitative: Strengths and difficulties questionnaire (SDQ) and beck youth inventory (BYI‐II) pre and post intervention to evaluate effectiveness of intervention at improving mental health and self‐concept.	Evaluation of intervention using case series analysis	Study length: 5 weeks Frequency of sessions: Weekly half day sessions (10 am‐1 pm)

Abbreviations: EHCP, Education, Health and Care Plan; FS, Forest School; FSA, Forest School Association; nr, not reported; PATH, Professional Association of Therapeutic Horsemanship International; PRUs, Pupil Referral Units; ScR, Scoping review; UWIST, University of Wales Institute of Science and Technology; WEMWBS, Based on 7‐item Warwick Edinburgh Mental Wellbeing Scale.

^a^ Only data for samples close to the defined age range (10–19 years) were included and reported in this table. (Several studies also reported findings for younger children, but these were not charted).

Most samples were at the younger end of the 10–19 years age‐range. Two studies recruited specifically NYP (Friedman et al., 2024; Tonks et al., 2025), while all other studies reported recruitment of pupils with needs more broadly categorised as behavioural difficulties, poor mental health or SEMH. Pupil setting was split between mainstream schooling (*k* = 4 secondary; *k* = 2 upper primary) and specialist schooling (*k* = 5). Eight were UK based, one was an Australian study, and six were situated in areas of low socio‐economic status.

There was variability across studies in study type, aims and methodology. All studies are classifiable as exploratory, in that they were either not designed for, or not sufficiently powered for, generalisability. All relied exclusively (*k* = 6), or heavily (*k* = 3), on self‐report measures (including interview, surveys/scales, and diaries), six used qualitative semi‐structured interviews, with two including practitioner/parent‐carer interviews. More than half of the studies utilised elements of pre‐post within‐participants design (*k* = 6). Finally, over half of the studies set out to evaluate the acceptability of the intervention, which often constituted the study's main focus, rather than primarily exploring mental health impact on individuals. Six different mental health and wellbeing measures were reported from the four studies reporting quantitative measures (see Table 3). At a superficial level, the six studies reporting qualitative analyses used thematic analysis, with one using qualitative description (Saggers & Strachan, 2016). Further detail on key descriptive characteristics of individual studies can be provided on request.

### Types of nature‐based education (review objective 1)

As shown in Table 3, the evidence in this review predominantly reported on FS interventions (*n* = 5), with four other studies focused on an environmental education initiative, a Social and Therapeutic Horticulture (STH) project, an Equine Facilitated Learning (EFL) initiative, and a bushcraft intervention.

Six studies reported on temporary programmes of varied length (single session to year‐long weekly programmes), while three were established programmes within school curriculums. All took place within the school day. Three provisions were within mainstream schooling, five were classifiable as specialist provision (special schools; a Special Education Unit within a mainstream school; and Pupil Referral Units), and one examined both mainstream and specialist schooling. The multi‐session programmes comprised weekly or monthly sessions over 3 months or more. Seven studies took place in natural environments, away from school grounds, with two on school premises.

In most papers, a basic description of session content and practice was provided. All programmes foregrounded promotion of social‐emotional wellbeing. Supplementary aims included indirect wellbeing (e.g., resilience, nature‐connection, self‐confidence, and self‐regulation); skills competence; and ‘green careers’ (careers in nature, conservation and environmental sectors). Therapeutic relationship‐building was inherent to many. Six studies specified that the projects were led by qualified and certified practitioners which included Level 3 qualified Forest School practitioners, certified Horticulturists, and qualified Equine Facilitated Learning practitioners. Adult to child ratios, where reported, were between 1:2 and 1:4.

### Mental health impact of nature‐based education (review objective 2)

Across all studies, the impact of NBE on the mental health and wellbeing of adolescents with a variety of neurodevelopmental needs was assessed as favourable, although study foci tended to be on acceptability of the intervention and wellbeing factors rather than mental health per se (see Table 4). All studies reported positive change across social, emotional and/or behavioural aspects and each concluded that the programme of interest had potential to improve the wellbeing and/or mental health of a range of young people.

**TABLE 4:** Summary table of analyses and findings from included studies.

Study	Nature of mental health outcomes	Reporting on mental health/wellbeing findings^a^	Nature‐based education components proposed as agents of change	Summary of author conclusions on mental health^a^
Chiumento et al. (2018)	Direct mental health measures and indirect factors associated with wellbeing.	MWIA: Most notable positive change was reported for deduced themes of *emotional wellbeing* and *self‐help.* Some evidence of raised priority given to mental health. Impact: In all schools, positive change for: emotional wellbeing; social networks/relationships; belief in own capabilities/self‐determination; sense of belonging; feeling involved; knowledge/skills to make healthy choices; and having a valued role. In 2/3 schools: Ability to understand/function socially. Importance: Ranked increase across schools for self‐help. In 2/3 schools: Belonging; activities bring people together; and ability to understand/function socially). Wellbeing check cards: Reported as non‐significant, with positive ‘trends’ for older ages and worsened scores for younger ages (potential confoundment). Qt‐Ql triangulation: Indication of positive trends in pro‐social and emotional factors.	Authors conject that multiple components may interact: ‐ Improved connection to others and place through quality of engagement and interaction opportunities ‐ Potential for natural environment to provide more creative and collaborative play opportunities. ‐ Time in green space: Stress reduction theory (Ulrich 1983) and attention restoration theory (Kaplan & Kaplan, 1989).	Authors concluded: ‐ Social therapeutic horticulture has the potential to improve wellbeing/mental health in young people with social, emotional and behavioural difficulties. ‐ Intervention and measures were consistent with Five Ways to Wellbeing framework.
Friedman et al. (2024)	Indirect elements and experiences considered to contribute to wellbeing.	Five SDT‐shaped themes linkable to mental health/wellbeing: ‐ Excitement and freedom of being beyond four walls ‐ Forest School affords opportunities for positive development ‐ Feelings regarding nature and Forest School are conditional and subject to change ‐ Rituals are important for all but must be tailored ‐ Attitudes of adults help or hinder sessions Experiences were described as highly diverse and generally positive (but not always). Forest School assessed as psychologically beneficial in that it was found to support all three tenets of SDT for most pupils.	Multiple autism‐related agents mentioned: ‐ Altered expectations and environment: Variety, novelty, freedom, autonomy (intrinsic motivator), acceptance of movement, adventure etc. ‐ Environment and activities provide prompts and space for play; peer‐relating; confidence‐building; nature connection; adult focus on child interests; self‐regulation, sensory needs and overcoming fears. ‐ Forest School rituals are valued.	‐ Forest School reported as positive for autistic pupils in that it promoted play; self‐direction: social, physical and practical skills development; and met psychological needs. ‐ Forest School routines, adult input and peer conflict moderated experiences. ‐ SDT was judged as a useful framework.
Garip et al. (2021)	Direct wellbeing measure and exploration of wellbeing experiences.	Five a priori themes (shaped Likert scales, survey questions and analysis): *wellbeing, confidence, nature connection, involvement,* and *understanding*. Quantitative: Significantly improved ratings described for all five teen survey questions: *confidence* was most improved and *wellbeing* least improved). Statistical values not reported for teen programme. Qualitative: Limited reporting on wellbeing factors for teen ages: for *Wellbeing*, a child's positive reaction is described (fun, not boring); for *confidence*, child's readiness for work and reduced fear is mentioned.	Unclear: None appeared specified for the teen sample.	‐ Across child and teen groups, a beneficial impact indicated for wellbeing, nature connection, engagement, self‐confidence and awareness after single structured session in green space.
Manner et al. (2021)	Direct wellbeing understandings and indirect elements.	Four hybrid‐derived themes: Mental wellbeing and resilience Community and social interaction Learning Perception of Forest School. Findings directly relevance to mental health: Mental wellbeing and resilience: Improvements noted in positive affect (happy/calm); confidence; social relations; and emotional literacy. *Community and social interaction*: Enhancement in social skills; family relations; emotional intelligence; community engagement and feeling included was generally reported.	Mental wellbeing/resilience: ‐ Space to learn to manage emotions and social interaction in peer group scaffolded by adults ‐ More opportunities to develop friendships and confidence. ‐ Forest School leads: supportive chats and conflict resolution ideas. ‐ Calm setting away from usual home/school stressors. Perception of Forest School: ‐ Compared with classrooms, outdoors represented freedom.	‐ Overall positive impact on teen social‐emotional wellbeing (possibly beyond sessions) and supported life skills (resilience, social relations, and managing problems) ‐ Forest School has potential as an early intervention for teen mental health.
Roe and Aspinall (2011)	Direct mood/wellbeing scale; plus, measure of indirect wellbeing contributor.	Mood: Where significant effects were noted, benefits from Forest School outperformed school, and were higher for ‘poor’ compared with ‘good’ behaviour groups, as follows: *Energy*: significant main effect difference of setting (*F* = 9.65, df = 1, *p* = 0.007, *ES* (partial eta) = 0.391); and a significant difference between behaviour groups (*F* = 9.48, df = 1, *p* = 0.008, *ES* = 0.387). *Stress*: Borderline main effect difference of setting (*F* = 4.44, df = 1, *p* = 0.052, *ES* = 0.229) and significant difference between groups (*F* = 5.45, df = 1, *p* = 0.034, *ES* = 0.267). *Hedonic tone*: significant main effect difference of setting (*F* = 9.99, df = 1, *p* = 0.007, *ES* = 0.416); borderline between groups (*F* = 4.49, df = 1, *p* = 0.052, *ES* = 0.243). *Anger*: significant main effect difference of setting (*F* = 6.65, df = 1, *p* = 0.020, *ES* = 0.293) only. Personal projects reflection (within‐group difference): No main effects of setting were detected. For Forest School only, trends in positive direction for *project support* and *project efficacy* subscales were described.	Mention of possibility that nature settings are conducive to better management of negative behaviours. N.B. Study did not set out to test components of FS and did not separate out effects of Forest School programme from environment.	‐ Forest School environments can be beneficial for pupils' mood (and may benefit goal reflection), with potentially greater effect for those with behaviour concerns. ‐ Extends theory on nature‐based restoration.
Saggers and Strachan (2016)	Indirect elements and experiences considered to contribute to wellbeing.	Two pre/post matching themes: One related to mental health (*Resilience*): All students expected (and confirmed) support in resilience‐building and social‐emotional wellbeing. All cited improved confidence; most mentioned improved social‐emotional regulation/coping, and stress management; and some improved communication; focus; and perseverance. Pupils felt intervention benefits might support them with school.	Reflections on possible agents: ‐ Insight on own and others' behaviours, emotions and needs gained from learning to read equine reactions. ‐ Developing trust and respect ‐ Working on goals as a group ‐ Acquiring horsemanship skill.	‐ Indication that Equine‐Facilitated learning supports social‐emotional development, resilience, engagement and connection.
Tiplady and Menter (2021) and Tiplady (2018)	Exploring wellbeing directly, and indirect elements considered to contribute to wellbeing.	Four themes outlined mostly positive wellbeing experiences: *engagement and enjoyment; relationships and interactions with others; perception of self;* and *beyond* Forest School (see right). ToC model theorised Forest School pathways to emotional wellbeing, with supporting evidence claimed as: ‘substantial’ for: improved interaction with peers/adults; and persistence/success with tasks. (‘Some evidence’ for: reduced anxiety; improved context‐appropriate behaviours; more awareness/sensitivity to others' emotional needs; and developing positive self‐narratives. N.B. Secondary school model data reported from Tiplady, 2018 (refers to same study). Emotional literacy: did not reach significance.	‐ *Enjoyment and engagement*: Talk variously linked benefits to setting: space/freedom; nature connection/relaxation; physical games; social aspect; enjoying learning; different to school. ‐ *Relationships and interactions*: More/better social/friendship opportunity; holistic approach (not curriculum‐led); high staff ratios; team‐work and child‐choice (intrinsic motivation). ‐ *Perception of self*: Positive self‐narratives linked to: nature of tasks; time for reflection; and group‐based social support.	‐ Individualistic, needs‐based, nature of benefits was key finding, which authors linked to child‐led focus of FS. ‐ Wellbeing benefits indicated for all pupils (and in‐line with proposed ToC). ‐ ToC model had utility in mapping steps to wellbeing and to evaluate FS.
Tiplady and Menter (2021)	Exploring wellbeing directly (and indirect elements considered to contribute to wellbeing).	Two themes ‐ *Physical environment* and *Learner‐centred pedagogy* ‐ highlighted positive development across domains including wellbeing, sense of self and managing trauma, anxiety and upset. ToC model: ‘Substantial evidence’ category assigned to improved comfort/skills interacting with others and improved ability to persevere with tasks etc. ‘Some evidence’ category claimed for develops positive self‐narratives.	‐ *Physical environment:* Benefit linked to the outdoors (calming, attention‐restoration, nature connection, and space to resolve conflict), and play opportunities (physicality, sensory feedback, resilience‐building). ‐ *Learner‐centred pedagogy:* Benefit linked to freedom to self‐direct (challenging for some), low stress, positive opportunities for imaginative play, risky/anxiety‐facing learning, scaffolded and unstructured socialising, and skills acquisition.	‐ Forest School's outdoor environment and learner‐centred pedagogy support psychological, physical, cognitive and social development. ‐ Positioned in contrast to lesser opportunities and distractions (e.g. screens) in usual home/school life.
Tonks et al. (2025)	Direct mental health measures.	Quantitative: BYI measure—all participants rated themselves as ‘above average’ pre intervention, and post intervention all but one were above average. Two made statistically significant improvements. SDQ measure—three participants were below clinical threshold pre and post intervention. Three improved from clinical to non‐clinical range (two of whom made statistically significant improvements). One deteriorated from non‐clinical to clinical range.	Author reflects that it may be the environment rather than the intervention that leads to improvements in wellbeing (outdoor setting, being outdoors, group process, gaining resilience skills and better coping mechanisms).	Reasonable and cautious interpretation of data suggests participation was associated with maintenance and improvement in wellbeing in the short term.

^a^ Only data concerning samples close to the defined age range (10–19 years) were included in the ScR and reported in this table (unless stated otherwise). As outlined in Table 3, two references reported on the same study, and information were extracted from both references together to answer the research objectives and are hence listed as one ‘study’ in this results table.

Roe and Aspinall (2011) measured mood using the UWIST4 14‐item Mood Adjective 2 Checklist (Matthews et al., 1990), and identified improved scores after a single FS session. Similarly, Garip et al. (2021) described improvements in wellbeing ratings after a single session, and Tonks et al. described statistically significant improvements in mental health and self‐concept following a 5‐week intervention (Tonks et al., 2025). Most studies did not restrict consideration to direct wellbeing factors but also explored constructs considered indirectly relatable to wellbeing (such as confidence, nature connection, or sense of control). Several wellbeing aspects reoccurred in the qualitative studies, including claims of improved emotional wellbeing; social wellbeing/functioning; self‐determination; and sense of belonging. Chiumento et al. (2018) reported the most notable changes in Mental Wellbeing Impact Assessment priorities following Social and Therapeutic Horticulture. All studies reported an increase in positive impact, with some indication of raised mental health priority relative to physical health. Looking specifically at FS, Manner et al. (2021) noted improved positive affect and emotional literacy in young people's talk; and Tiplady and Menter identified some evidence for reduced anxiety (Tiplady & Menter, 2021) and managing trauma/anxiety/upset (Tiplady & Menter, 2022). Saggers & Strachan (2016) described improved emotional regulation and coping, and resilience‐building was a key theme (noted in other studies along with task perseverance (Tiplady & Menter, 2021; Tiplady & Menter, 2022; Tonks et al., 2025)).

Other commonly‐cited improvements included change in social wellbeing and functioning for example increase in perceived impact on social networks and relationships (Chiumento et al., 2018); promotion of social development (Friedman et al., 2024); improved social skills, family relations and emotional intelligence (Manner et al., 2021); improved communication (Saggers & Strachan, 2016) and evidence relating to interaction with peers/adults and sensitivity to others' emotional needs (Tiplady and Menter, 2021, 2022). Improved self‐determination (or beliefs around capability, self‐confidence, self‐direction, self‐narratives or self‐help) were also recurring themes mentioned within the context of post‐intervention improvement (Chiumento et al., 2018; Friedman et al., 2024; Manner et al., 2021; Saggers & Strachan, 2016; Tiplady and Menter, 2021, 2022). Similarly, Garip et al. (2021) identified confidence as representing the largest effect size change across their five programme‐based objectives. Finally enhanced sense of belonging or feeling involved themes were highlighted by both Chiumento et al. and Manner et al. (Chiumento et al., 2018; Garip et al., 2021).

Tiplady and Menter, 2021, 2022 and Friedman et al. also reported some individual negative experiences such as peer conflict, and Chiumento et al. (2018) noted the potential for reduced wellbeing although this was not robustly supported in their data. Tonks et al. (2025) indicated that one participant deteriorated from non‐clinical to clinical range, and Friedman et al. (2024) concluded from their qualitative data that FS does not represent a cure‐all for the issues that autistic people face in schools, describing FS sessions as requiring navigation of challenges for pupils and practitioners. Similarly, Tonks et al. (2025) emphasised that there is a need to understand individual differences prior to intervention delivery.

### Nature‐based education components as agents of change (review objective 3)

Most studies did not aim to identify or separate out NBE components as agents in wellbeing, and none aimed to test causation. However, all studies speculated on potential components that elicited mental health or wellbeing impacts, evidenced by examples from qualitative or observational data, or in some cases, psychological theory (see Table 4). In mapping steps to improved emotional wellbeing, Tiplady and Menter's coproduced Theory of Change (ToC) provides systematic insight of identification of nature‐based mechanisms. The model primarily cites components within the individual (e.g., “CYP become more comfortable interacting…”), with some FS components included (e.g., “presence of a potentially less‐threatening environment”) and an emphasis on group work, reflection, and self‐direction. This model was one stream of a larger ToC developed by the authors, which included consideration of the provision and environment as well as the individual's wellbeing.

Affordances of the natural outdoor environment and the nature/ethos of educational activities were commonly reported across studies. Both elements were frequently cited as providing more ‘space’ and ‘prompts’ for social, emotional and behavioural development in contrast to typical school environments, and many identified both factors as providing enhanced opportunities for: social interaction and friendships (Chiumento et al., 2018; Friedman et al., 2024; Manner et al., 2021; Tiplady and Menter, 2021, 2022); imaginative and/or collaborative play (Chiumento et al., 2018; Friedman et al., 2024; Tiplady and Menter, 2021, 2022); self‐regulation/managing emotions and confidence‐building (Friedman et al., 2024; Manner et al., 2021); developing connection to nature and/or place (Chiumento et al., 2018; Friedman et al., 2024; Tiplady and Menter, 2021, 2022), and resilience‐building with enhanced opportunity to overcome fears and learn from risk‐taking (Saggers & Strachan, 2016; Tiplady & Menter, 2022; Tonks et al., 2025). Both overarching components were also linked repeatedly with notions of mental/physical freedom, space and autonomy, as well as altered expectations and acceptance of behaviours (such as allowing more movement and physicality).

Outdoor environments were independently seen as better at meeting sensory needs, providing attention restoration, stress reduction and calm, and were valued for variety, novelty and adventure. Components highlighted as specific to pedagogy included the learner‐centred, needs‐based, holistic approach (enabling of self‐direction and intrinsic motivation (*k* = 2); focus on practical skills and perseverance (*k* = 3); inclusion of reflection time, routines and unstructured time (*k* = 2); goal‐based group activities (*k* = 3); and the higher ratios and practitioner expertise (in supporting individuals, social interactions, conflict resolution and group activity where needed) (*k* = 3). Friedman et al. (2024) also suggested components and moderators specifically valuable to NYP, including adult ability to give quality time to a child's special interests.

## DISCUSSION

This systematic ScR provides a detailed presentation of the current peer reviewed evidence base exploring the role of NBE in the mental health and wellbeing of neurodivergent adolescents (NYP). It addresses three objectives, each of which are discussed below. Overall, it demonstrated a paucity of studies in this area, which are primarily qualitative and small‐scale.

In relation to objective one, five NBE types were identified. There was clear variety across approach, organisational sector/structure, regulatory status and funding models, and a diversity of practice in terms of content, pedagogy and delivery. Each practice however, appeared to adhere to the ethos, standards, training and best practice relevant to each NBE type. This heterogeneity within the NBE sector is in keeping with findings of a multi‐agency DEFRA‐funded overview of NBE in the UK which found it to be highly fragmented (Burt et al., 2016), but a key commonality across NBE types was that all positioned emotional wellbeing and positive youth development as central to their rationale and practice, reflecting current research and practice recommendations (Jani et al., 2023).

In addressing objective two, all studies linked NBE to direct and indirect mental health and wellbeing benefits for pupils with a variety of neurodevelopmental concerns, across social, emotional, psychological and/or behavioural domains, but only three authors reported using robust validated measures to elicit evidence of positive changes (Roe & Aspinall, 2011; Tiplady & Menter, 2021; Tonks et al., 2025). Further, there was overlap in terms of the type of wellbeing benefits described, and little focus on mental health relative to wellbeing. This overall finding of potential wellbeing benefit in a NYP population aged 10–19, is consistent with findings from key stakeholders working within the general school population: Natural England's (Burt et al., 2016) overview found 92% of 125 schools (including secondaries) agreed that a programme of learning in the natural environment had conferred positive health and wellbeing impacts for pupils (reported by teachers, teaching assistants and support staff), and implicated a similar set of wellbeing benefits to that found in this review. It also concurs with systematic reviews of wellbeing benefits in younger populations related to access to green spaces (McCormick, 2017), nature engagement (Gill, 2014) and FS (Garden & Downes, 2023).

The limited evidence base identified presents challenges in synthesising robust evidence of mental health and wellbeing impact. However, it is of note that several authors identified patterns of individual wellbeing benefits, and benefits are potentially differentiated by emotional or behavioural need; for example, one study found greater benefit experienced by those with reported ‘negative’ compared to ‘good’ behaviour (Friedman et al., 2024; Roe & Aspinall, 2011; Tiplady & Menter, 2021). This raises the possibility that NBE may be most valuable for those experiencing greater challenge within typical school environments; the cohesive findings across the multiple methods in the evidence base, in addition to other more recent findings (Loose et al., 2024), potentially indicates this as a fruitful line of further enquiry.

Addressing objective three, the included studies described a range of NBE components that may confer positive mental health impact for NYP, however, none of the studies set out to specifically test or distinguish different components beyond (Tiplady & Menter, 2022) ToC. Two overarching, interlinked drivers were apparent across NBE types: the impacts of being in/with nature and the NBE pedagogy. Within these, NBE was conceptualised as providing space and prompts for enhanced opportunities for a variety of beneficial mechanisms to occur, and amongst these, social, cognitive, emotional/regulatory, motivational, and psychological factors were repeatedly mentioned. This reflects previous research findings that have reported on access to nature being a protective factor for children's mental health (Chawla, 2015; Gray et al., 2025), highlighted stress reduction and attention restoration mechanisms that occur from spending time in nature (Bratman et al., 2012), and emphasised that regular schooling in the outdoors can promote positive outcomes for children in social, academic, physical and psychological dimensions (Becker et al., 2017; Miller et al., 2021).

The largely qualitative designs reported in these studies provide some rich insights of NBE experiences (the focus on young person report is a strength of the literature), and some quantitative analysis has provided important initial insights and direction. However, major limitations exist pertaining to the mental health benefit that NBE may hold for NYP. Crucially, no controlled trials were identified and none of the included studies were designed to, or capable of, inferring causation or generalisability. This echoes the findings of Garden & Downes (2023) systematic review of FS literature in the UK which highlighted how this field of research is highly context‐specific and lacking in generalisability and applicability. Similarly, all but one of the studies in this review are UK based, despite there being examples of NBE being used worldwide (Cumming & Nash, 2015; Dabaja, 2025; Harper, 2017; Hofman‐Bergholm, 2023; Knight, 2016; Takriti et al., 2020).

Further limitations of the evidence base include the use of bespoke, unvalidated scales (Garip et al., 2021) or methods not previously tested on youth populations (Chiumento et al., 2018). Longer‐term follow‐up assessments were not utilised and there were variation and ambiguity in the duration of programmes examined. Two of the studies measured effects following one‐off sessions (Garip et al., 2021; Roe & Aspinall, 2011), despite existing NBE pedagogy suggesting that it should be a long‐term process with regular rather than one off sessions (Becker et al., 2017; Waite & Goodenough, 2018). Furthermore, no studies appeared to involve any private school provision, parent‐carer fee‐paying NBE or home‐educated populations. With an estimated 81,000 children currently home educated, and disproportionately likely to be classed as having SEND (The Centre for Social Justice, 2022), this represents a significant research gap. Finally, the included samples are under‐representative of upper teenage years, with almost all involving populations aged under 16. Along with the need for controlled trials, these all represent valid and useful next steps for research in this area.

Whilst the findings in this review largely point to primarily positive outcomes, documenting potential adverse effects would be valuable and more longitudinal studies are required to support a robust evaluation of NBE as a preventative public health intervention. It cannot be gleaned from this review whether the paucity of published findings, and particularly the limited reporting of robust quantitative analyses, arises from inherent difficulties in examining NBE and its impacts on NYP, or simply reflects that this is a relatively new research strand for which NYP have not yet been a focus. Previous reviews have explored wellbeing outcomes in relation to natural environments, but with various populations and not necessarily with a focus on time spent in nature for educational purposes (Gill, 2014; Gray et al., 2025; Holland et al., 2025; Tillmann et al., 2018). The sector has experienced a rapid increase in provision since the 1990s (Leather, 2018), and FS specifically has been prominent in the UK for over 30 years (Cree & McCree, 2012; Knight, 2016). There has been a recognised demand for NBE within and outside of schools (Bryant, 2021; Burt et al., 2016), and the numbers of NYP experiencing emotional distress at school is high (Connolly et al., 2023). The lack of peer‐reviewed research articles assessing impacts of NBE on mental health beyond small‐scale exploratory findings is therefore noteworthy and perhaps reflects a lack of integration of health and education research more broadly, despite there being acknowledgement of health outcomes being intrinsically linked to education experiences, given the proportion of their lives that young people spend in education settings (The Lancet Public Health, 2020).

Whilst isolating wellbeing benefits within any complex intervention is challenging (Blodgett et al., 2022), controlling for the huge variety and diversity within NBE makes it particularly difficult for research to evidence benefits. NBE encompasses a variety of programmes (Garden & Downes, 2023), with an open flexible pedagogical model approach, content fundamentally adapted to child need, child‐led direction (Cree & McCree, 2012; Leather, 2018) and in‐the‐moment experiential opportunities afforded by the environment (Cree & Robb, 2021; Knight, 2016). As such, there is no set curriculum, and different practitioners may take very different approaches to content and extent of formal learning. The sector is generally unregulated, and not subject to any inspection process beyond some internal local authority and school quality assurance processes (Department for Education, 2025).

Nonetheless, recent developments involving larger‐scale controlled studies of NBE within broader populations are promising. The Open Skies project (Loose et al., 2024) recently reported on a cluster randomised controlled trial of nature‐based intervention in Canada, finding some evidence of positive impact, but only for those aged 10–12 years that were experiencing poorer mental health symptoms (in line with above mentioned potentially differentiating benefits of NBE). Similarly, Humber Teaching NHS Foundation Trust and the University of York are currently undertaking a feasibility cluster randomised controlled trial of school‐based FS on mental health in primary aged children (Armitt et al., 2025), and a more recent review of NBE on mental health in secondary schools has emphasised benefits such as reduced anxiety and increased resilience but highlighted the lack of focus on cognitive outcomes and moderate quality of evidence (Lorimer et al., 2026). The findings from our review add to this rapidly growing evidence base and provide scope for further defined research in this area to promote alternative models of education which better support young people's mental health.

### Strengths and limitations

A strength of this review is the rigorous search and screening process that was followed, and the deliberately broad description of NBE and associated search terms which allowed relevant literature related to a wide range of interventions and settings to be identified, as well as the specific focus on neurodivergent young people. Whilst these search parameters were relevant for this review, it is however limited by the specific focus on education as opposed to therapeutic practices. It is possible that widening the eligibility criteria to include therapeutic and adventure‐based interventions or approaches, and search terms related to wider range of specific neurodevelopmental disorders (in addition to Autism and ADHD) and mental health conditions, may have elicited valuable findings in the field which weren't identified with the process implemented in this review.

## CONCLUSION

This review has demonstrated the heterogeneity in existing research exploring types of NBE, and their impact on the mental health in NYP. Studies were generally small‐scale, exploratory primary research studies, largely reporting qualitative findings. The literature is characterised by a lack of consistent approach or theory as well as limited investigation of the specific components of NBE that confer mental health impacts and thus lacked generalisability. However, all included studies reported positive impacts on mental health for NYP overall and this review provides a structured categorisation of the key research and elements of NBE that are relevant to mental health and wellbeing that can provide a useful framework or springboard for future examination.

Within the context of increasing nature deficit and worsening mental health for young people (Mckinlay et al., 2024) and as NYP are disproportionately more likely to experience school distress and attendance difficulties (Fielding et al., 2025), it is imperative that further evidence is developed to understand alternative educational models that can appropriately promote positive mental health for this population as well as support learning. Current research points to the positive impact that NBE can have on mental health, but more work is needed to robustly evaluate NBE as an alternative educational model and identify key components that may confer mental health benefits for NYP specifically.

## AUTHOR CONTRIBUTIONS

**Helen Eke**: Conceptualization; investigation; funding acquisition; writing—original draft; methodology; writing—review and editing; validation; formal analysis; project administration; data curation; supervision. **Suzie Holt**: Writing—original draft; writing—review and editing; formal analysis; data curation; investigation; validation; visualization. **Helen Dodd**: Writing—review and editing; supervision. **Faraz Mughal**: Writing—review and editing; supervision. **Eleanor Shaw**: Investigation; project administration. **Abigail Emma Russell**: Writing—review and editing; supervision; conceptualization; funding acquisition.

## CONFLICT OF INTEREST STATEMENT

The authors declare no conflicts of interest.

## ETHICAL CONSIDERATIONS

Ethical approval was not sought for this review as it consisted secondary data analysis of publicly available published data, and was not a requirement of the lead institution.

## Data Availability

The data that support the findings of this study are available from the corresponding author upon reasonable request.
